# Non-contact monitoring of agitation and use of a sheltering device in patients with dementia in emergency departments: a feasibility study

**DOI:** 10.1186/s12888-020-02573-5

**Published:** 2020-04-15

**Authors:** Lisa Kroll, Nikolaus Böhning, Heidi Müßigbrodt, Maria Stahl, Pavel Halkin, Birgit Liehr, Christine Grunow, Borjana Kujumdshieva-Böhning, Christian Freise, Werner Hopfenmüller, Wolfgang Friesdorf, Maria Jockers-Scherübl, Rajan Somasundaram

**Affiliations:** 1 Charité - Universitätsmedizin Berlin, corporate member of Freie Universität Berlin, Humboldt-Universität zu Berlin, and Berlin Institute of Health, Emergency Department Campus Benjamin Franklin, Hindenburgdamm 30, 12200 Berlin, Germany; 2 iDoc - Institut für Telemedizin und Gesundheitskommunikation GmbH & Co. KG, Berlin, Germany; 3 Oberhavel Kliniken, Department of Psychiatry, Psychotherapy and Psychosomatic Medicine, Hennigsdorf, Germany; 4 Technical University of Berlin— Institute of Psychology and Ergonomics, Berlin, Germany; 5 HCMB – Institute for Health Care Systems Management Berlin eG, Berlin, Germany

**Keywords:** Dementia, agitation, Geriatric care, Non-contact monitoring, Emergency department

## Abstract

**Background:**

Agitation is common in geriatric patients with cognitive impairment, e.g. in persons with dementia (PWD), who are admitted to an emergency department (ED). It might be a first sign of upcoming delirium and is associated with a higher risk of an unfavorable clinical course. Hence, monitoring of vital signs and enhanced movement as indicators of upcoming agitation is essential in these patients during their stay in the ED. Since PWD rarely tolerate fixed monitoring devices, a novel developed non-contact monitoring system (NCMSys) might represent an appropriate alternative.

Aim of this feasibility study was to test the validity of a NCMSys and of the tent-like “Charité Dome” (ChD), aimed to shelter PWD from the busy ED environment. Furthermore, effects of the ChD on wellbeing and agitation of PWD were investigated.

**Methods:**

Both devices were attached to patient’s bed. Tests on technical validity and safety issues of NCMSys and ChD were performed at the iDoc institute with six healthy volunteers. A feasibility study evaluating the reliability of the NCMSys with and without the ChD was performed in the real-life setting of an ED and on a geriatric-gerontopsychiatric ward. 19 patients were included, ten males and nine females; mean age: 77.4 (55–93) years of which 14 were PWD. PWD inclusion criteria were age ≥ 55 years, a dementia diagnosis and a written consent (by patients or by a custodian). Exclusion criteria were acute life-threatening situations and a missing consent.

**Results:**

Measurements of heart rate, changes in movement and sound emissions by the NCMSys were valid, whereas patient movements affected respiratory rate measurements. The ChD did not impact patients’ vital signs or movements in our study setting. However, 53% of the PWD (7/13) and most of the patients without dementia (4/5) benefited from its use regarding their agitation and overall wellbeing.

**Conclusions:**

The results of this feasibility study encourage a future controlled clinical trial in geriatric ED patients, including PWD, to further evaluate if our concept of non-contact measurement of vital signs and movement combined with the “Charité Dome” helps to prevent upcoming agitation in this vulnerable patient group in the ED.

**Trial registration:**

ICTRP: “Charité-Dome-Study - DRKS00014737” (retrospectively registered).

## Background

Admission to a hospital’s emergency department (ED) due to an acute medical incident is a threatening situation for geriatric patients with cognitive impairment, especially in persons with dementia (PWD) [1]. The overstimulating surroundings often lead to more agitation and increase the probability of complications such as the development of delirium, the risk of falls and injuries as well as getting lost [2]. Furthermore, older PWD are more likely to develop a delirium and show a higher in-hospital mortality compared to older patients without any cognitive impairment (10.8 vs. 6,6% in a large retrospective observational study of unscheduled admissions of patient aged ≥75 years, most of them via the ED) [3].

PWD’s lack of understanding and therefore missing cooperation makes their therapy a difficult task to fit in hospital routine, especially in the ED [2]. Often, the only possibility to deal with PWD is to administer sedatives with sometimes hazardous side effects or their fixation with belts [4]. These conditions result in longer stays, higher mortality rates and increased functional decline for PWD in hospital compared to patients without dementia [5, 6]. As a consequence, there is a need for special care in terms of ED architecture [7, 8] or devices to protect PWD from sensory overload and devices to detect and monitor upcoming agitation, possibly indicated by a change of vital signs, e.g. heart rate and respiratory rate [9]. As monitoring of vital signs usually requires the use of adhesive electrodes or the attachment of sensors, which PWD tend to tear off their body due to discomfort [2], the development of new technical tools for non-contact monitoring of vital signs is necessary. Thus, wearable devices have been developed [10] that have to be worn continuously, which is difficult in a hospital situation with frequent change of gown and the need of quick access to the patients’ chest in the case of an emergency. A visible bedside device used e.g. for sleep apnoea studies [11] might be removed or damaged by agitated patients.

Many attempts have been made to prevent worsening of a beginning agitated state in PWD. There is evidence for preventive efficacy of non-pharmacological treatments [12], though there are no in-hospital studies, especially in the ED, which mirrors the difficulty to implement them [13]. Therefore, we developed and tested a novel non-contact monitoring system (NCMSys) to monitor vital signs, such as heart and respiratory rate, combined with monitoring of movement and sound emissions, without the necessity of a direct patient contact. Furthermore, a tent-like device, the “Charité Dome” (ChD), was designed, aimed to shelter PWD from sensory overload of the busy ED environment. Both systems were intertwined from the very beginning of their development and were attached to the patient’s bed and evaluated in terms of validity of measurements and effects on patient’s wellbeing and agitation in the real-life setting of an ED and a geriatric-gerontopsychiatric ward. Here, we present first results of this feasibility study.

## Methods

### Development of the “Charité dome” (ChD) and the non-contact monitoring system (NCMSys)

An innovation project of the School of Design Thinking, Potsdam, Germany, and the Charité Universitätsmedizin Berlin aimed to improve the complex situation of PWD in EDs which should be achieved by the development of a novel system for data collection or analysis and for shielding of agitated patients. This led to the ChD’s first prototype. The ChD is a cover for the head section of a hospital bed, resembling a baby carriage’s convertible top. It was designed in cooperation with the Gesslein GmbH, a baby carriage company, the iDoc Institute for Telemedicine and Health Communication, an expert for telemedicine, and the department of Human Factors Engineering and Ergonomics from the Technical University of Berlin.

To evaluate the effects of the ChD on upcoming agitation, mirrored by changes in vital signs, increased movement and sound emissions (e.g. screaming), we developed a NCMSys combining specialized software and

**Fig. 1:**
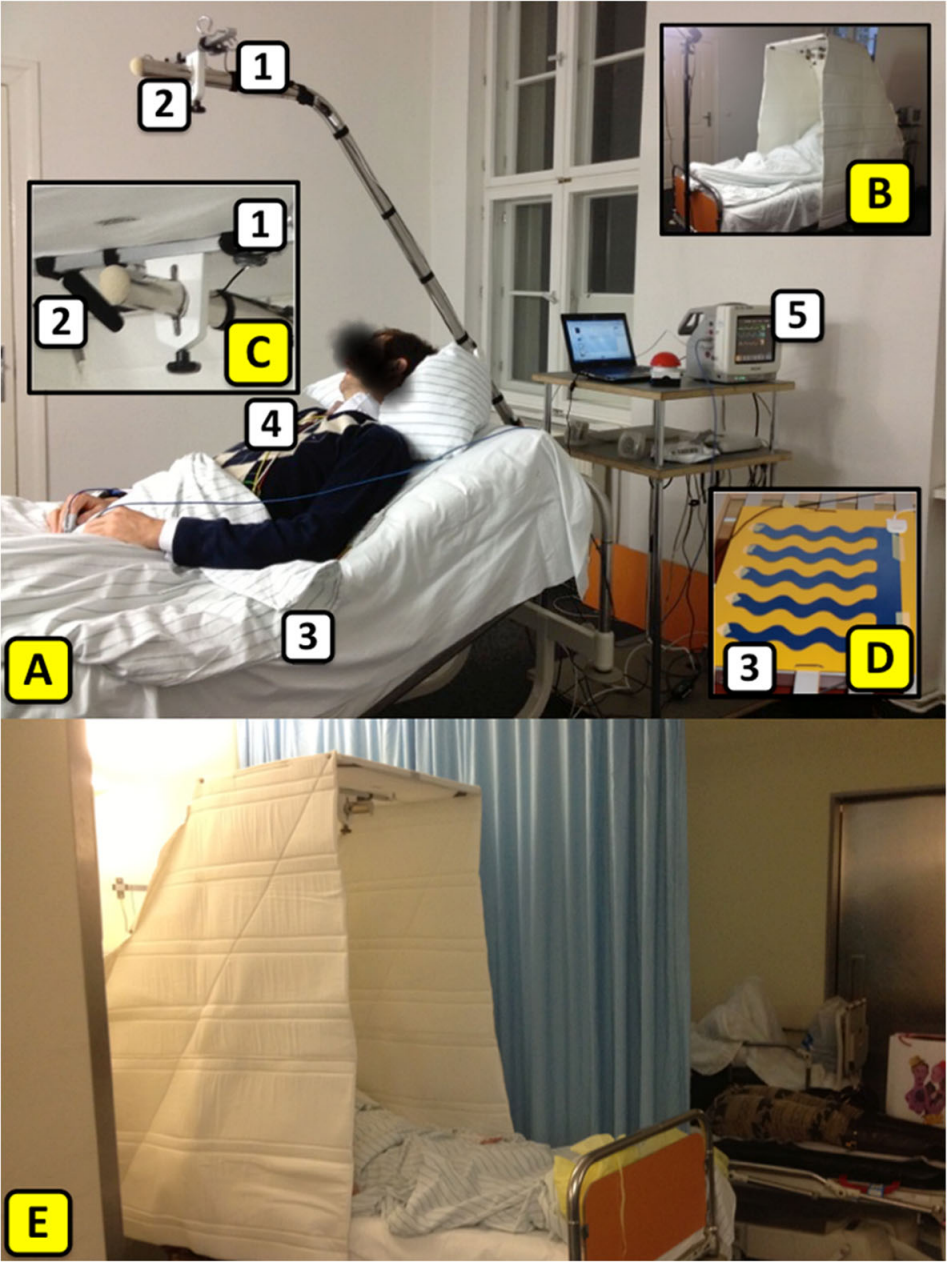
Arrangement of the non-contact measurement system (NCMSys) and the Charité Dome (ChD). **a** NCMSys without and **b** with the ChD during the feasibility study: (1) visual and (2) acoustic sensor (enlarged illustrated in (**c**); **d** sensor mat and (3) its location (4) ECG electrodes; (5) monitor. **e** NCMSys and ChD during the feasibility study in the ED

1. a visual sensor (Mini-Webcam, Conrad Electronics SE, Hirschau, Germany) detecting changes in movement (Figure 1a1),
2. an acoustic sensor (ME32, Olympus Imaging Europa GmbH, Hamburg, Germany) installed to record potential verbal expression (Figure 1a2),
3. a sensor mat (SafeBed IP system, Emfit® Ltd., Vaajakoski, Finland) with no direct contact to the test person integrated into the mattress measuring heart rate, respiratory rate and changes in movements (Figure 1a3).

**Fig. 2:**
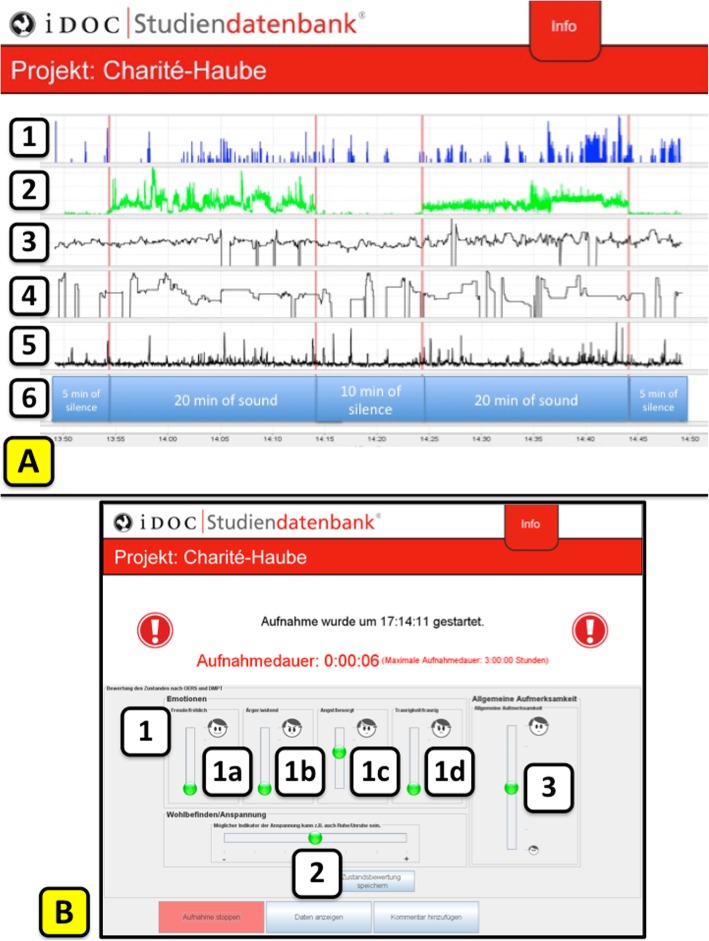
Exemplary screenshots of the simulation software. **a** Screenshot study non-contact monitoring. (1) number of motions registered by visual sensor; (2) sound recording; (3) heart rate per minute; (4) respiratory rate per minute; (5) activity registered by sensor mat; (6) timeline. **b** Screenshot of the “user-friendly touch-screen interface” for rating (1) emotions: (1a) joy; (1b) anger; (1c) fear; (1d) sadness (only one emotion out of the four could be selected.); (2) wellbeing/stress; (3) general alertness during the feasibility study in the ED and the geriatric-gerontopsychiatric ward

All vital signs, movements and sounds were recorded simultaneously. The installed video camera took 24 pictures / second and recorded only differences of each picture which were expressed as a signal. Of note, the camera did not record “photographic images”, only the pixel changes per frame (24fps) were detected. The same applies for the recorded audio signals. Only sound intensities in decibel (dB) and not e.g. spoken words were detected, recorded and displayed as a graph over time (Fig. 2a).

Specialized software (iDoc-Studiendatenbank, iDoc-Institut, Potsdam/Berlin, Germany) collected and visualized the recordings of all connected devices (Fig. 2a).

To evaluate the validity of the NCMSys and safety issues of the ChD, pre-tests with healthy volunteers were performed, followed by a first feasibility study combining the ChD and NCMSys in a clinical setting, aiming to test if there was evidence for a future and well-designed, controlled clinical trial and thus, depending on the results, its use in future clinical routine.

### Validity of the non-contact monitoring system (NCMSys) alone and in combination with the “Charité dome” (ChD)

The validity of the presented system was tested at the iDoc institute. Six healthy volunteers (age 22–82 years) were included as test persons. They were connected to a monitor (model MP5, Philips, Amsterdam, The Netherlands) to measure heart rate and respiratory rate (“standard monitoring”). Additionally, the new approach of a combined non-contact monitoring was tested. The study was conducted in a room equipped with constant lighting conditions and a standard hospital bed (Fig. 1a) over a period of two hours per test person. To assess the influence of the ChD on the NCMSys, the ChD was installed on the bed (Fig. 1b) after the first hour. Different sorts of sounds were played to provide a standardized acoustical surrounding and to test the reliability of the sound recording.

### Feasibility study in the ED and a geriatric-gerontopsychiatric ward

A feasibility study evaluating the effects of the ChD and its combination with NCMSys was conducted in the ED of the Charité Universitätsmedizin Berlin at the Campus Benjamin Franklin and the dementia-specialized geriatric-gerontopsychiatric ward of the Oberhavel Kliniken Hennigsdorf.

Nineteen patients were included, ten males and nine females. Patients in the ED as well as at the geriatric-gerontopsychiatric ward were investigated during the main working hours from Monday to Friday and mainly in the afternoon, the most busy time in the ED. Eight patients were included at the ED and eleven patients at the dementia-specialized geriatric-gerontopsychiatric ward. They had different acute medical problems. The mean age of the patients was 77.4 (55 to 93) years. Three patients of the ED and all eleven patients of the dementia-specialized geriatric-gerontopsychiatric ward were PWD. PWD inclusion criteria were age 55 years or older, a documented dementia diagnosis from the past medical history (especially patients in the ED) and/or by Mini Mental Status Examination and assessment by General Deterioration Scale (patients at the geriatric-gerontopsychiatric ward) as well as the patients’ or their legal guardians’ written consent. For patients without dementia, inclusion criteria were age 18 years or older and the written consent to take part in the study. Exclusion criteria were acute life-threatening situations (corresponding to Manchester Triage System “red”) or an acute risk of harm to patients themselves or others and a missing ability for consent.

In addition, and complementary to the NCMSys an interface for a standardized assessment of the effects of the ChD by staff, based on the Observed Emotion Rating Scale [14] and the Dementia Mood Picture Test [15], was used during the feasibility study (Fig. 2b). Furthermore, staffs’ comments were registered. In case of conflicting results, staffs’ comments were rated higher than the assessment based on the interface.

The feasibility study in the ED was performed during daytime and in the more standardized patients’ rooms or the observation room of the geriatric-gerontopsychiatric ward. To measure the effects of the ChD on patients’ vital signs, movements and sound emission, monitoring was performed during two hours per patient. During the first hour, the patients were lying in a standard hospital bed equipped with the NCMSys. During the second hour, the bed was additionally equipped with the ChD prototype (Fig. 1e).

After each one-hour monitoring with and without ChD the patients without dementia and – if present – the relatives of the PWD in the ED were asked to fill out a standardized form based on the Observed Emotion Rating Scale regarding the patients’ state of mood.

### Patient and public involvement

Patients’ priorities, experience, and preferences did not affect the development of the research question and outcome measures. Patients were not involved in the design of the study nor in the recruitment to and conduct of the study. Results of the study will be freely available after their publication.

### Statistical analysis

Spearman correlations were performed to analyse differences between measurements of heart rate and respiratory rate by standard monitoring and NCMSys. Effects of the ChD on heart rate, respiratory rate, the activity values, and the number of motions were analysed by t tests. All statistical calculations were performed with the SPSS Software (Version 23.0).

## Results

### Validity of non-contact monitoring of heart and respiratory rate, sound and movement in healthy test persons

In all six healthy test persons there was a clear correlation for the first (R^2^ = 0.874; without ChD) and the second (R^2^ = 0.608; with ChD) hour of heart rate monitoring between the reference monitor and the sensor mat. In contrast, the sensor mat did not show reliable measurements of the respiratory rate (correlation of R^2^ = 0.840 in the first, R^2^ = 0.062 in the second hour), *please see* Additional file 1*. This is a phenomenon also known from other attempts for a device-assisted monitoring of vital signs* [16, 17].

The acoustic sensor showed a valid registration of the different standardized sounds with only little perturbation and artefacts (Fig. 2a, line 2).

Visual sensor and sensor mat registered changes in movement. Figure 2a gives a representative example of the registered changes in movement curves of the visual sensor (number of motions, line 1) and the sensor mat (activity, line 5) during a one-hour period with a healthy test person lying in bed. Depending on the kind of changes in movement, e.g. just moving hands over or under the covers, registration by the visual sensor was not always paralleled by registration of the sensor mat, pointing to complementarity of both systems. This was seen even more pronounced when examining patients.

### Non-contact monitoring of heart and respiratory rate, changes of movement and sound emissions in ED patients and patients on a geriatric-gerontopsychiatric ward

From 19 patients one patient had to be excluded after the monitoring started due to missing cooperation, resulting in 18 patients to be evaluated. Throughout the test of the ChD, the NCMSys monitored reliably with only little perturbations and dropouts.

### Effects of the Charité dome measured by the NCMSys and assessed by staff, patients themselves and relatives

In the specific study setting changes in sound emissions and heart rate measured by the NCMSys did not show significant differences between the one hour spent with and without the ChD (see Additional file 1).

But analysis of the assessment by the attending staff in the ED and geriatric-gerontopsychiatric ward, patients themselves and relatives pointed to positive effects on patients’ mood by assessing emotion, wellbeing, and alertness (results are summarized in Table 1).

**Table 1:** Effects of the Charité Dome on patients’ mood rated by staff, patients themselves and relatives (summarized). Assessment is based on the Observed Emotion Rating Scale and the Dementia Mood Picture Test. Detailed comments of the staff are shown in Additional file 1

	Amelioration	No change	Deterioration
Emergency Department – Patients without Dementia			
	4	–	1
Emergency Department – Patients with Dementia			
	2	1	–
Geriatric-gerontopsychiatric Ward			
	5	1	4

Amelioration was perceived by higher levels of joy, general alertness and wellbeing. Some patients snuggled into the panels of the ChD, others were calmer in their physical agitation. Deterioration was mirrored by decreases of mood, alertness and wellbeing. Being restricted and having limited sight were reasons to dislike the ChD (deterioration).

## Discussion

### Principal results

We here present a novel concept combining a new non-contact measurement system of vital parameters and movement coupled with a tent-like device, aimed to shelter PWD from the busy environment in the ED. The combined system showed valid results in the real-life setting of an ED and on a geriatric-gerontopsychiatric ward.

### Non-contact measurement system (NCMSys)

The NCMSys recording of heart rate, changes in movement and sound emissions were valid in all settings when test persons or patients were lying in bed.

With regard to extended clinical tests of the NCMSys/ChD, the obtained correlation values of ~ 0.6–0.8 for heartrate monitoring between the reference monitor and the sensor mat needs a further improvement. Nevertheless, this is a proof of a new concept. Taking into account that heart rate measurement in agitated patients might be even more difficult in terms of accuracy by established systems which are attached to the patients, our system works valid in the ED setting.

This was not the case for respiratory rate. The correlation of respiratory rate differed strongly between the two test hours due to test persons’ and patients’ movements. The latter is a known problem, either when other systems are used, such as camera photoplethysmography for heart rate monitoring [16] or sensor mats for continuous measurements, e.g. in intensive care units, unless the patients are asleep or sedated [17]. Thus, we cannot recommend non-contact measurement of respiratory rate in this setting being in line with other findings, that to date, non-contact monitoring of respiratory rate is not well-engineered for applications in general [11]. However, monitoring of respiratory rate is essential, particularly in the ED. New technical systems were published just recently [18, 19] but need further evaluation in the setting “PWD in the ED”.

Changes in vital signs may – to some extent – indicate upcoming or manifestation of agitation, e.g. in intensive care patients [9, 20]. Due to the mentioned limitations of non-contact monitoring of respiratory rate, we therefore complemented our system by a visual and an acoustic sensor. The tested sensor mat and visual sensor complemented each other for monitoring changes in movement: The sensor mat covered pressure-full whole-body movement like turning, while the visual sensor also covered smaller movements like fiddling fingers above the blanket. The latter may mirror increasing nervousness and agitation and was not always detected by the sensor mats’ monitoring alone or by solely measuring vital signs. The acoustic sensor should be evaluated in further studies to complement the system for its use in the clinical setting.

From the point of practicability, our NCMSys has the advantage of an easy and quick installation (and deinstallation) on a standard hospital bed, a precondition if it was used in busy EDs.

### “Charité dome” (ChD)

More than half of the PWDs and most of the patients without dementia lying beneath the ChD experienced beneficial effects in terms of emotion, wellbeing and alertness. Thus, the ChD might be suitable counteracting upcoming agitation. Since “hard-fact” data such as vital signs are very rare for this patient group, comparable studies showed, that for measuring the success of non-pharmaceutical implementations PWD’s experience and carers’ assessment could be more meaningful than such data sets [21]. We here confirm this assumption with our data regarding the ChD, which are mainly based on the assessment of health care professionals, relatives and patients themselves.

During the two-hour setting of our feasibility study (1 h without, 1 h with the ChD), no changes of vital signs were detected by the NCMSys neither in the healthy test persons nor in the patient group. This might be due to the short time period of measurement and especially due to the heterogeneity of the studied patients. The patients on geriatric-gerontopsychiatric ward were mobile, whereas ED patients were mainly lying in bed because of acute medical problems. Future studies should focus on the latter patient group, which then should be monitored by our system for the whole time of their stay in the ED. However, for some PWDs the ChD even had an agitation promoting effect. Therefore, a close observation at the beginning of the lying-beneath-the-ChD-phase in future studies will be needed to identify those patients.

A major advantage of the ChD compared to e.g. bringing the PWD to a separate room is the limited amount of space it requires. Especially in the ED the available space for beds is often limited. Bed curtains also provide a certain private sphere, though the sheltering effect of the ChD is much more distinctive. Furthermore, the ChD mounted to the bed allows to move PWD in the ED without disturbing there immediate “surrounding”. However, a respective experimental comparison should be considered in follow-up studies.

### Limitations of the study

Only a few patients were included in our feasibility study, therefore the population is too small to include a detailed statistical analysis. Further, the test times were rather short and were restricted to daytime. The assessments of amelioration and deterioration were estimated by experienced nursing staff in a non-standardized manner. Therefore, an individual “human factor” cannot be excluded. Furthermore, even the few patients in the ED were studied under more “quiet” conditions, although extrinsic factors causing further stress for patients – beside their acute medical problem – could not be excluded completely. Thus, there might be a bias in the estimated grade of wellbeing of patients in our setting. Having in mind a potential translation of our approach into the clinical routine, more patients and a less artificial study setting (e.g. a real-life, but defined ED-surrounding during the whole time of patient’s stay in the ED) need to be considered in a future controlled clinical trial.

## Conclusions

The combined concept of the presented non-contact measurement system to detect heart rate, changes in movement and sound emission complemented by the ChD might be suitable to detect upcoming agitation, e.g. due to delirium, and to prevent further deterioration in older patients with cognitive impairment, including PWD, bound to bed in the ED. This concept may help to ameliorate quality of care, e.g. by less demand for sedating medication, and may result in shorter in-hospital stays of these patients [22]. Furthermore, this may lead to cost reductions for the public health sector [23] without reduction of quality of care. Future studies will focus on further technical and design development of the NCMSys and the ChD, respectively, including a valid non-contact detection of respiratory rate. The results of our feasibility study support the initiation of a controlled clinical trial to evaluate if our system ameliorates level of care of older patients with cognitive impairment, especially PWD, in an ED. Such a trial must take the legal and ethical issues into account, and finally, it must be emphasized, that a technical solution only can complement, but never replace personal care of this very vulnerable group of patients in the ED.

## Supplementary information

**Additional file 1: Figure S1.** Validity of non-contact monitoring of heart and respiratory rate, sound, and movement in healthy test-persons. Linear correlation (A) for the first (correlation of R^2^ = 0.874) and (B) the second hour (R^2^ = 0.608) of HR monitoring between the reference monitor and the SM in all six healthy test persons. Correlation for the measurement of RR (C) in the first (R^2^ = 0.840) and (D) in the second hour (R^2^ = 0.062). **Figure S2.** Effects of the ChD on vital parameters of patients. Vital parameters of patients lying in bed were measured for one hour without the ChD followed for one hour with the ChD. Differences for the (A) heart rate, (B) the respiratory rate, (C) the activity values and (D) the number of recorded motions were analyzed by multiple t-tests using the Holm-Sidak method, with alpha = 5.000%. **Table S1.** Evaluation interface based on parameters of OERS and DMPT.

## Data Availability

The datasets used and/or analysed during the current study are available from the corresponding author on reasonable request.

## Abbreviations

AS: Acoustic sensor
ChD: Charité dome
ED: Emergency department
NCMSys: Non-contact monitoring system
PWD: Person(s) with dementia
