# Supplementary material for: Non-contact monitoring of agitation and use of a sheltering device in patients with dementia in emergency departments: a feasibility study
Source: BMC Psychiatry. 2020 Apr 15;20:165. doi: 10.1186/s12888-020-02573-5 (PMC7161155; doi:10.1186/s12888-020-02573-5)
Supplement: Supplementary file 1 — Additional file 1: Figure S1. Validity of non-contact monitoring of heart and respiratory rate, sound, and movement in healthy test-persons. Linear correlation (A) for the first (correlation of R2 = 0.874) and (B) the second hour (R2 = 0.608) of HR monitoring between the reference monitor and the SM in all six healthy test persons. Correlation for the measurement of RR (C) in the first (R2 = 0.840) and (D) in the second hour (R2 = 0.062). Figure S2. Effects of the ChD on vital parameters of patients. Vital parameters of patients lying in bed were measured for one hour without the ChD followed for one hour with the ChD. Differences for the (A) heart rate, (B) the respiratory rate, (C) the activity values and (D) the number of recorded motions were analyzed by multiple t-tests using the Holm-Sidak method, with alpha = 5.000%. Table S1. Evaluation interface based on parameters of OERS and DMPT. [file 12888_2020_2573_MOESM1_ESM.docx]

**Additional files**

**Kroll *et al.* “**NON-CONTACT MONITORING OF AGITATION AND USE OF A SHELTERING DEVICE IN PATIENTS WITH DEMENTIA IN EMERGENCY DEPARTMENTS: A FEASIBILITY STUDY**”**

**Additional file 1:**

**Figure S1**

**A**


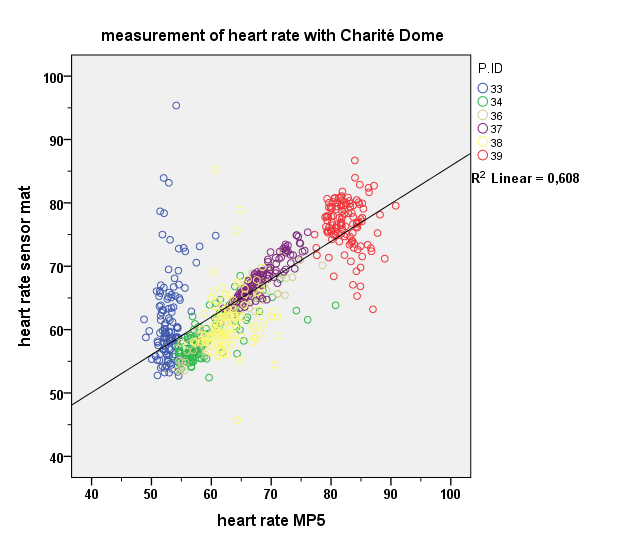


**0.608**

**B**


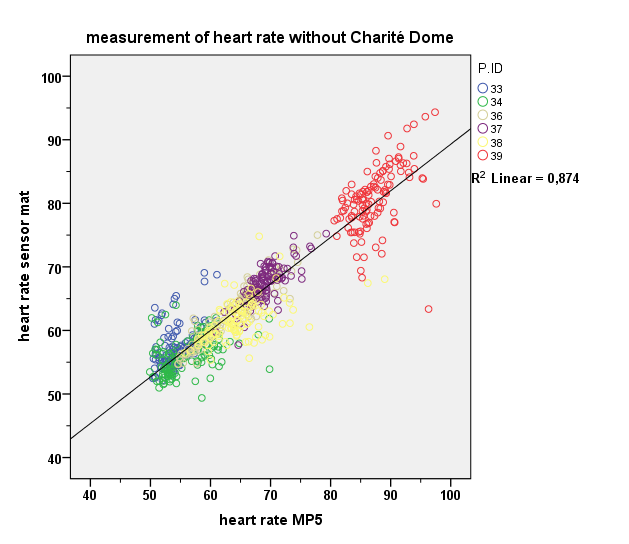


**0.874**

**C**


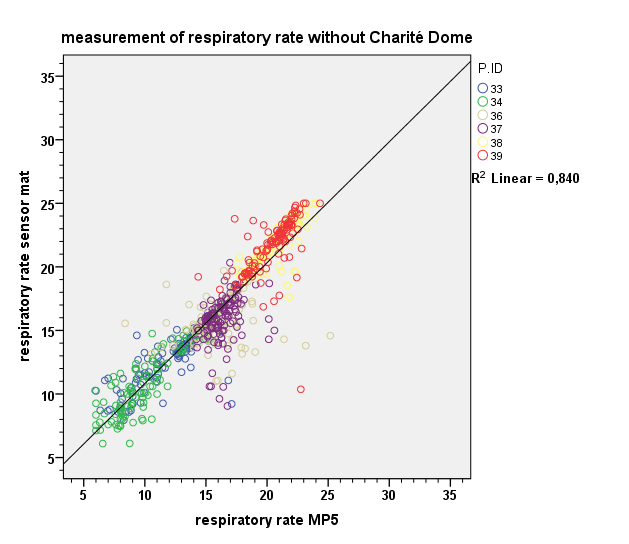


**0.840**

**D**


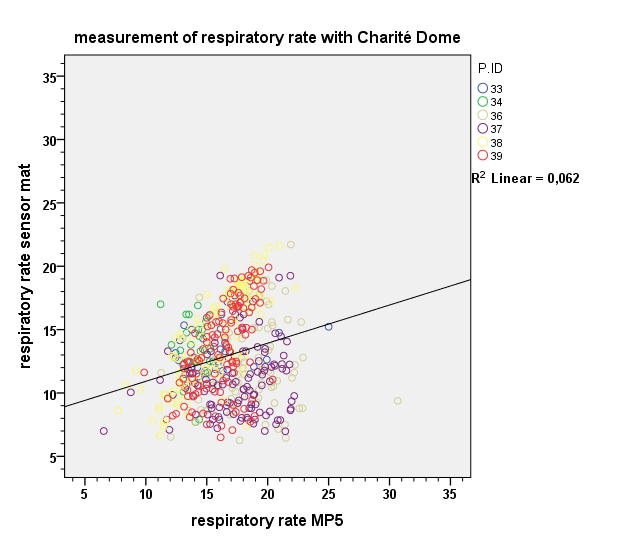


**0.062**

**Figure S1: Validity of non-contact monitoring of heart and respiratory rate, sound, and movement in healthy test-persons**. Linear correlation (**A**) for the first (correlation of R^2^=0.874) and (**B**) the second hour (R^2^=0.608) of HR monitoring between the reference monitor and the SM in all six healthy test persons. Correlation for the measurement of RR (**C**) in the first (R^2^=0.840) and (**D**) in the second hour (R^2^=0.062).

**Additional file 2:**

**Figure S2**

**A Heart rate (BPM)**


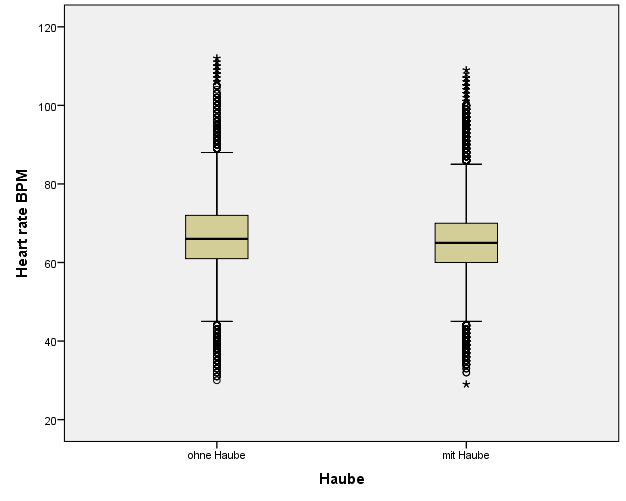


***P*>.05**

**With ChD**

**Without ChD**

**B Respirationrate (per minute)**

***P*>.05**


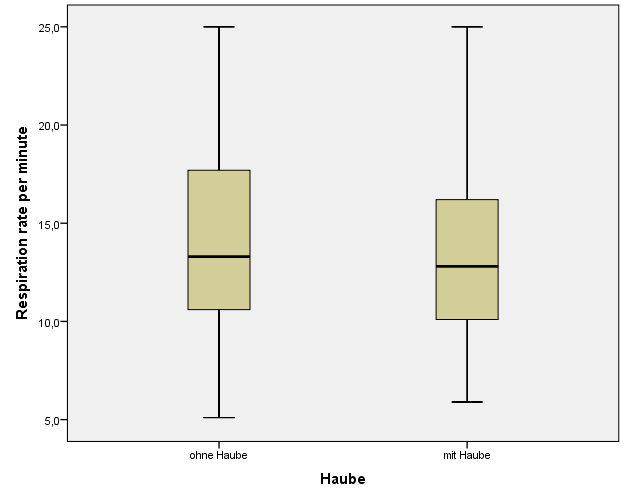


**Without ChD**

**With ChD**

**C Activity value**

***P*>.05**


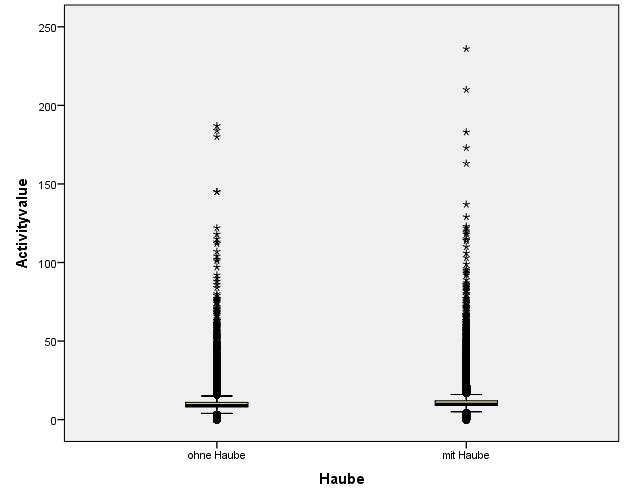


**With ChD**

**Without ChD**

**D Number of motions**


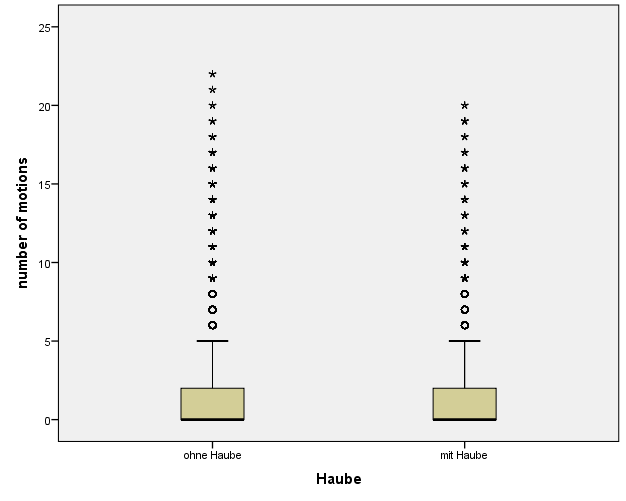


***P*>.05**

**With ChD**

**Without ChD**

**Figure S2: Effects of the ChD on vital parameters of patients.** Vital parameters of patients lying in bed were measured for one hour without the ChD followed for one hour with the ChD. Differences for the (A) heart rate, (B) the respiratory rate, (C) the activity values and (D) the number of recorded motions were analyzed by multiple t-tests using the Holm-Sidak method, with alpha=5.000%.

**Additional file 3:**

**Table S1: Evaluation interface based on parameters of OERS and DMPT**

**Dementia Patients:**

| **Pat-ID** | **Situation** | **Emotion (1-5): happy, sad, anxious, angry** | **General alertness** | **Wellbeing/**  **Stress** | **Trend** | **Additional comments of observing staff** |
| --- | --- | --- | --- | --- | --- | --- |
| 50/2001 | Before ChD | joy (4) | 3 | 3 |  |  |
|  | After ChD | joy (1) | 3 | 2 | Deterioriation | Pt. generally agitated |
|  |  |  |  |  |  |  |
| 51/2002 | Before ChD | n.s. | 3 | 1 |  |  |
|  | After ChD | n.s. | 3 | 0 | Deterioriation | Pt. often calls, screams and complains in general; tries to look past the ChD |
|  |  |  |  |  |  |  |
| 52/2003 | Before ChD | n.s. | 4 | 4 |  |  |
|  | After ChD | n.s. | 3 | 3 | Amelioration | Pt. likes the ChD |
|  |  |  |  |  |  |  |
| 53/2004 | Before ChD | joy (3) | 4 | 3 |  |  |
|  | After ChD | joy (3) | 4 | 4 | Amelioration | Pt. much calmer after the ChD is mounted. Cuddling to the side canvas of the ChD Permanent nurse judges ChD for that pat. beneficial, observing his cuddling inside it |
|  |  |  |  |  |  |  |
| 54/2005 | Before ChD | n.s. | 3 | 4 |  |  |
|  | After ChD | n.s. | 4 | 4 | Deterioriation | Pt. does not like the ChD |
|  |  |  |  |  |  |  |
| 55/2006 | Before ChD | joy (1) | 4 | 4 |  |  |
|  | After ChD | joy (2) | 4 | 5 | Amelioration | Pt. is more relaxed and likes the ChD |
|  |  |  |  |  |  |  |
| 56/2007 | Before ChD | joy (1) | 3 | 4 |  |  |
|  | After ChD | joy (1) | 3 | 3 | Deterioriation | Pt. agitated and does not like the ChD |
|  |  |  |  |  |  |  |
| 58/2009 | Before ChD | joy (1) | 3 | 3 |  |  |
|  | After ChD | joy (1) | 5 | 5 | Amelioration | Pt. calmer under the ChD |
|  |  |  |  |  |  |  |
| 59/2010 | Before ChD | fear (1) | 4 | 3 |  |  |
|  | After ChD | fear (1) | 5 | 5 | Amelioration | Agitated pt. |
|  |  |  |  |  |  |  |
| 60/2011 | Before ChD | sadness (1) | 4 | 5 |  |  |
|  | After ChD | n.s. | 3 | 3 | No change | Pt. very cooperative |
|  |  |  |  |  |  |  |
| 45/1005 | Before ChD | joy (5) | n.s. | n.s. |  |  |
|  | After ChD | joy (5) | n.s. | n.s. | No change |  |
|  |  |  |  |  |  |  |
| 47/1006 | Before ChD | n.s. | 2 | 2 |  |  |
|  | After ChD | n.s. | 4 | 3 | Amelioration |  |
|  |  |  |  |  |  |  |
| 48/1007 | Before ChD | n.s. | 1 | 3 |  |  |
|  | After ChD | joy (3) | 5 | 4 | Amelioration | Wife talks very positively about ChD |
|  |  |  |  |  |  |  |
| **Non-Dementia Patients:** | | | | | | |
| **Pat-ID** | **Situation** | **Emotion (1-5): happy, sad, anxious, angry** | **General alertness** | **Wellbeing/**  **Stress** | **Trend** | **Additional comments of observing staff** |
|  | After ChD | joy (1) | 3 | 3 | Amelioration | Pt. feels more relaxed |
|  |  |  |  |  |  |  |
| 41/1002 | Before ChD | joy (5) | 3 | 3 |  |  |
|  | After ChD | joy (5) | 4 | 1 | Deterioration | Pt. is afraid of getting too warm in the summertime |
|  |  |  |  |  |  |  |
| 42/1003 | Before CD | n.s. | 4 | 3 |  |  |
|  | After CD | joy (1) | 4 | 3 | Amelioration | Pt. is relieved and likes the lighting conditions |
|  |  |  |  |  |  |  |
| 43/1004 | Before CD | joy (3) | 4 | 3 |  |  |
|  | After CD | joy (4) | 4 | 4 | Amelioration | Pt. likes better privacy protection and better lighting conditions |
|  |  |  |  |  |  |  |
| 49/1008 | Before CD | joy (3) | 3 | 3 |  |  |
|  | After CD | joy (4) | 5 | 4 | Amelioration | Pt. calls the ChD cozy and comfortable |

Abbreviations: ChD, Charité Dome; Pt., patient; OERS, Observed Emotion Rating Scale; DMPT, Dementia Mood Picture Test
